# Caveolin-1 expression in benign and malignant lesions of the breast

**DOI:** 10.1186/1477-7819-5-110

**Published:** 2007-10-03

**Authors:** Cornelia Liedtke, Christian Kersting, Horst Bürger, Ludwig Kiesel, Pia Wülfing

**Affiliations:** 1 Department of Obstetrics and Gynecology, University of Münster, Albert-Schweitzer-Str. 33, 48149 Münster, Germany; 2 Gerhard Domagk Institute of Pathology, University of Münster, Domagstr. 17, 48149 Münster, Germany; 3 Institute of Pathology, Husener Str. 46 a 33098 Paderborn, Germany

## Abstract

**Background:**

Caveolin-1 is thought to have an important impact on both signal transduction and mediation of intracellular processes. Furthermore, it has been suggested that Caveolin-1 may contribute to certain steps of carcinogenesis in various types of cancer. We examined the potential clinical relevance of Caveolin-1 in normal, benign and malignant breast tissue specimens.

**Methods:**

Using tissue microarray (TMA) technology cases of invasive breast cancer, DCIS, benign breast disease (i.e. fibroadenoma, sclerosing adenosis, ductal hyperplasia and radial scar) and normal breast tissue were evaluated for Caveolin-1 expression. Immunohistochemical staining with an anti-Caveolin-1-antibody was performed. Staining intensity was quantified semiquantitatively. In invasive lesions staining results were correlated with clinical and pathological data.

**Results:**

No Caveolin-1 expression was observed in epithelial cells of normal breast tissue (n = 5), benign breast disease (n = 295) and DCIS (n = 108). However, Caveolin-1 expression was found in 32 of 109 cases of invasive breast carcinomas (29.4%). Caveolin-1 expression in invasive breast cancer could neither be correlated with survival parameters such as overall or disease-free survival nor with established clinical and pathological markers.

**Conclusion:**

In this study we demonstrated expression of Caveolin-1 in one third of invasive breast cancers. A significant increase in Caveolin-1 expression was observed comparing invasive breast cancer to both benign breast tissue and non-invasive breast cancer. Since inhibitors of Caveolin-1 signalling are available, targeting Caveolin-1 in breast cancer may represent a potential option for future breast cancer treatment.

## Background

Invasive breast cancer is still the most common female malignancy worldwide and more than 1 million women are diagnosed with breast cancer each year [1]. Caveolae are flask-shaped invaginations of the plasma membrane with an average diameter of 50–100 nm. The members of the Caveolin family comprise the essential protein compound of caveolae and stabilize the asymmetric distribution of lipids in this particular region [2]. Caveolin-1 has been found to interact with numerous proteins such as the heterotrimeric G-proteins [3], ha-ras, the members of the src-family of tyrosine kinases [4], and the endothelial nitrooxid-synthase (eNOS) [5]. Based on the formation of heterooligomeric complexes between Caveolin-1 and both integral membrane proteins and cytoplasmic signalling molecules, the Caveolin-signalling hypothesis has been established. It describes a process of compartmentalization of distinct signaling molecules exerting an important impact on cell signalling pathways by coupling activated receptors to secondary cellular effector systems [6]. NIH3T3 cells transformed by oncogenes such as v-abl- or h-ras show reduced or even complete absence of Caveolin-1-mRNA or -protein expression [7]. Hence, a tumour-suppressive function of Caveolin-1 has been suggested. However, in a significant number of tumour entities including carcinoma of the pancreas [8], squamous cell carcinoma of the lung [9], renal cell carcinoma [10], and carcinoma of the prostate [11], overexpression of Caveolin-1 has been described.

With regards to breast cancer only limited and conflicting data exists. Caveolin-1 has been reported to be downregulated in a number of human breast cancer cell lines as well as in tumours derived from transgenic rodents with breast cancer [12]. Loss of heterozygosity (LOH) at 7q31.1–7q31.2 has been shown to be a common event in breast cancer and the presence of a tumour suppressor gene had been suggested accordingly [13]. However, Caveolin-1 expression could not be shown to correlate with LOH at the *CAV-1 *locus [14]. Thus, the role of Caveolin-1 in breast cancer tumourigenesis and progression still remains ill-defined.

The aim of this study was to comprehensively examine expression of Caveolin-1 in different benign and malignant breast tissues, including DCIS and invasive breast cancer using tissue microarray (TMA) technology.

## Methods

### Patients

200 breast cancer specimens were obtained from patients primarily diagnosed with breast carcinoma, who underwent surgery at the Department of Gynaecology, University of Münster (Germany), between 1993 and 1995. The corresponding formalin-fixed paraffin-embedded tissue-specimens were obtained from the archives of the Gerhard-Domagk-Institute of Pathology (University Hospital Münster). The series of breast carcinomas previously has been characterized with respect to histopathological and clinical parameters and expression of ER, PR, HER2 and Mib-1 [15,16] (table 1). All of the 245 patients with invasive breast cancer were treated with therapeutic surgery (69 mastectomy and 155 wide local excision) and adjuvant anthracycline-based chemotherapy, and those with ER-positive tumours also received endocrine therapy. No neoadjuvant chemotherapy was performed. Mean disease-free survival (DFS) was 83 ± 3 months (95%-CI 77–90), mean overall survival (OS) 90 ± 3 months (95%CI 85–96).

**Table 1:** Characteristics of patients with invasive breast carcinomas

	**Parameter**	**n**	**%**
**Histologic type:**	infiltrating ductal	97	54.5
	lobular	40	22.5
	tubular	9	5.1
	mucinous	4	2.2
	medullary	4	2.2
	mixed-type	24	13.5
	unknown	22	

**pT-stage:**	pT1	76	42.7
	pT2	55	30.9
	pT3	13	7.3
	pT4	34	19.1
	pTx	22	

**pN-stage:**	pN0	94	54.3
	pN1	66	38.2
	pN2	13	7.5
	pN3	0	0
	pNx	27	

**cM-stage**	M0	156	87.7
	M1	22	12.3
	pMx	22	

**Grading:**	G1	16	9.0
	G2	98	55.1
	G3	64	35.9
	unknown	22	

**ER expression**	positive	105	62.9
	negative	62	37.1
	unknown	33	

**PR expression**	positive	77	46.4
	negative	89	53.6
	unknown	34	

**HER2expression**	positive	15	8.9
	negative	154	91.1
	unknown	31	

**MIB-1 expression**	< 20%	105	62.5
	≥ 20%	63	37.5
	unknown	32	

We also studied 200 cases of DCIS. All cases were classified according to the criteria outlined by Holland *et al*., based on nuclear grading and architectural features [17]. With respect to this classification, cases were graduated as low grade (n = 54), intermediate grade (n = 49), and high grade (n = 94). The median age of patients was 59 years (range 18–94 years) [18].

Furthermore, we included samples of normal breast tissue (n = 5) obtained from patients undergoing reduction mammoplasty, and a set of benign breast disease (n = 295) in the study. Benign lesions comprised cases of fibroadenoma (n = 167), sclerosing adenosis (n = 93), ductal hyperplasia (n = 33) and radial scar (n = 2).

#### Preparation of TMA

Routinely fixed paraffin-embedded tissue blocks containing tumours excised at the time of surgery were extracted from the files of pathology laboratories, and served as donor blocks for the TMAs. Tumour samples were arrayed in analogy to the procedure formerly described by Kononen *et al*., [19]. Briefly, for each sample three morphologically representative tissue areas were defined based on haematoxylin and eosin (HE)-stained sections. From each of these three areas, a tissue cylinder was punched out from the donor blocks using a precision instrument (Beecher Instruments) and transferred precisely into a new recipient paraffin block (20 × 35 mm). Each cylinder had a diameter of 0.6 mm.

#### Immunohistochemistry

3 μm sections from the TMA blocks were mounted on polylysine-coated microslides, dewaxed and rehydrated. For antigen retrieval, tissue slides were immersed in Reveal Emulgator (Biocarta, Hamburg, Germany) and boiled in a pressure cooker (103 kPa/15 psi for 5 min.). Subsequently, the sections were washed in Aqua dest. and Phosphate buffered saline (Sigma), and then subjected to Aurion-BSA-c10% (Aurion, Wageningen, Netherlands) in order to block unspecific binding-agents. This step was followed by overnight exposure (4°C) to the primary monoclonal mouse-IgG1-anti-Caveolin-1-antibody (BD Biosciences Pharmingen, Heidelberg, Germany; clone 2297; dilution: 1:200). This antibody has been used and validated previously by others [20,21]. After rinsing in PBS, H_2_O_2_-containing methanol (concentration: 0.6%) was applied to provide quenching of endogenous peroxidase activity, followed by incubation with the bridging goat anti-mouse-immunoglobulins conjugated with horseradish peroxidase (HRP)-labelled dextran polymer for one hour (DAKO Envision, HRP, Mouse). Final washing in PBS was then followed by visualization of the peroxidase enzyme using Vector SG Substrate Kit for HRP (Vector Laboratories, Burlingame, CA, USA) as well as nuclear counterstaining with haematoxylin. Positive staining of smooth muscle cells or endothelium, known to be abundant in Caveolin-1, served as positive control, while the omission of primary antibody served as negative control.

#### Microscopic analysis

**Figure 1:**
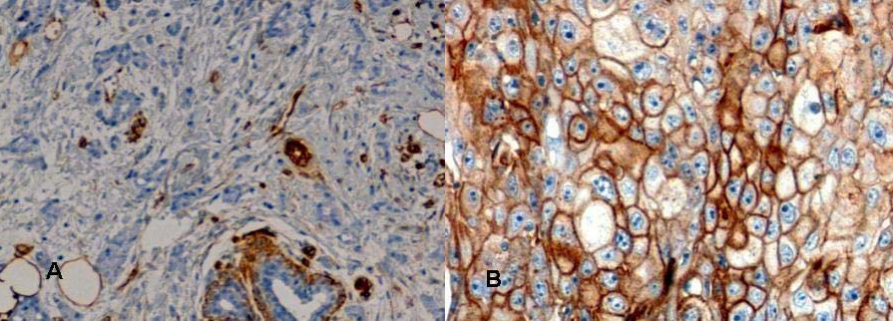
**Caveolin-1 expression in breast cancer samples**. A: Breast cancer sample without expression of Caveolin-1 in the epithelial tumor component. In contrast, expression of Caveolin-1 can be seen in myoepithelial cells as well as endothelial cells of entrapped vessels, serving as internal positive control (100× magnification). B: Breast cancer sample with strong expression of Caveolin-1 in the epithelial tumor component (400× magnification)

Caveolin-1 expression was then evaluated in a semiquantitative manner. Only membranous with or without cytoplasmic staining was considered specific, which is in concordance with previous studies [21]. Previous studies have also reported on the use of nonneoplastic endothelial cells as internal positive controls for immunohistochemical Caveolin-1 expression analysis [21]. Accordingly, in our study entrapped vessels served as internal positive control, revealing a positive staining for anti-Caveolin-1 antibody. Membrane staining was scored on a four-tired scale from "0" (no staining), over "1+" (weak staining), "2+" (moderate staining) to "3+" (strong staining) (Figure 1A and 1B). Depending on the staining procedure varying numbers of tissue cores were detached. Others did not contain sufficient numbers of tumour cells. Therefore, some cases could not be analyzed. In the case of more than one evaluable tissue probe, mean expression levels were obtained. Tumours assessed to show no Caveolin-1 expression at all (staining score 0) were defined as "Caveolin-1 negative", whereas weakly, moderately and strongly stained tissue cores were taken together as "Caveolin-1 positive". Evaluation of Caveolin-1 staining was performed in a blinded manner without knowledge of the assigned clinical data. Analysis of other prognostic and predictive factors such as ER-, PR-, HER2-, and MIB-1 expression was performed as described previously [16]. "MIB-1 positive" staining had been previously defined as > 20% of the cells showing MIB-1 expression [15,16].

### Statistical analysis

Statistical analysis of obtained data was performed using SPSS-Software. For 178 patients with primary invasive breast cancer sufficient survival data could be obtained. These patients were included in survival analysis. Kaplan-Meier curves for DFS and OS were generated and compared by log-rank test. Correlation analysis between Caveolin-1 expression and clinical and pathological data including information on tumour staging (TNM stage), histological grading, hormone receptor and HER2 status was performed using cross-tables applying χ^2 ^test.

## Results

### Caveolin-1 expression

Caveolin-1 expression could be determined in 109 of 200 cases of invasive breast cancers (54.5%). 32 cases of invasive breast cancer (29.4%) were found to be positive for Caveolin-1. Among these cases, 24 (22.0%) showed a weak, 6 (5.5%) a moderate, and 2 (1.8%) a strong staining (Table 2; Figure 1A and 1B).

**Table 2:** Immunohistochemical analysis of Caveolin-1 expression in primary human breast carcinomas

Caveolin-1 expression score (semiquantitative)	frequency (n)	percentage (%)
0	77	70.6
1	24	22.0
2	6	5.5
3	2	1.9

Total	109	100.0

negative ^§^	77	70.6

positive ^#^	32	29.4

^$ ^all tumours with a Caveolin-1 expression score of 0;

# all tumours with a Caveolin-1 expression score of 1 – 3

Caveolin-1 expression could be evaluated in 108 of 200 cases of DCIS (54.0%). Among these cases, none displayed a significant staining for Caveolin-1.

Caveolin-1 expression could be determined in 236 of 295 cases of benign breast disease (80.0%); in detail, 148 of 167 cases of fibroadenoma (88.6%), 65 of 93 cases of sclerosing adenosis (69.9%), 21 of 33 cases of ductal hyperplasia (63.6%) and both cases of radial scar (100%) could be evaluated. Among these cases, none showed significant expression of Caveolin-1 in the epithelial component of the benign disease.

Evaluation of normal breast tissue samples showed no expression of Caveolin-1 in breast epithelial cells. In myoepithelial cells of ducts and lobuli as well as in blood vessels, expression of Caveolin-1 was consistently found in normal breast tissue as well as both benign breast diseases and DCIS.

### Correlation studies

Correlation studies performed in the cases of invasive breast cancer showed that none of the clinical and pathological factors significantly correlated with Caveolin-1 expression, with the exception of positive correlation between Caveolin-1 expression and multifocality (p = 0.008) (Table 3). We further examined the distribution of Caveolin-1 expression among distinct histological subtypes of breast cancer. Correlation analysis regarding histological subtypes did not reveal a significant correlation of any of these with Caveolin-1 expression status.

**Table 3:** Correlation analysis of Caveolin-1 expression with clinical and pathological variables in primary breast cancer patients. Included are only cases in both Caveolin-1 expression status as well as clinical and pathological variable were available

clinical and pathological variables		Caveolin-1 positive/all tumours (%)	p (χ^2^-test)
ER	negative	15/41 (36.6)	0.204
		
	positive	16/64 (25.0)	

PR	negative	20/65 (30.8)	0.721
		
	positive	11/40 (55.0)	

HER2	negative	28/98 (28.6)	0.320
		
	positive	4/9 (44.4)	

Mib1	< 20%	15/39 (38.4)	0.143
		
	≥ 20%	17/68 (25.0)	

pT-stage	T1/2	22/80 (27.5)	0.476
		
	T3/4	10/29 (34.5)	

pN-stage	negative	18/60 (30.0)	0.981
		
	positive	14/47 (29.8)	

pM-stage	negative	29/94 (30.9)	0.391
		
	positive	3/15 (20.0)	

tumour grade	1	1/6 (16.7)	0.740
		
	2	17/59 (28.8)	
		
	3	14/44 (31.8)	

multifocality	no	19/83 (22.9)	0.008
		
	yes	13/26 (50.0)	

inflammatory	no	30/102 (29.4)	0.962
		
	yes	2/7 (28.6)	

lymphangiosis carcinomatosa	no	22/82 (26.8)	0.312
		
	yes	10/27 (37.0)	

### Survival analysis

**Figure 2:**
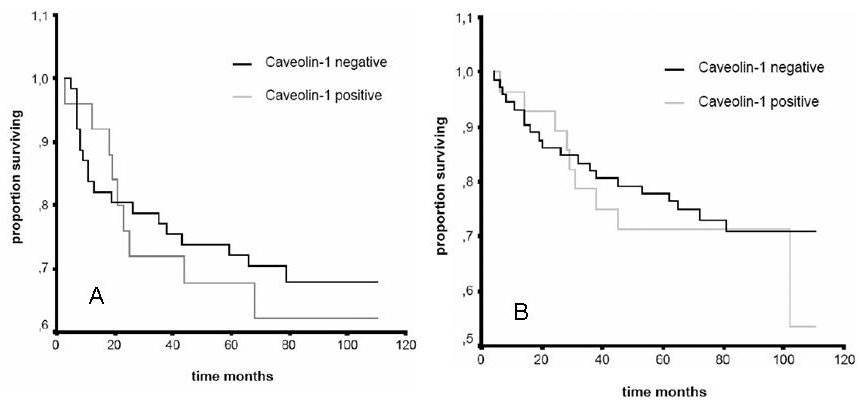
**Kaplan Meier estimates for OS and DFS stratified by Caveolin-1 expression**. A: Mean DFS in the Caveolin-1 positive and the Caveolin-1 negative group was 82 months (95% Confidence Interval 72–93 months) and 78 months (95%CI 62–95; log rank: p = 0.66), respectively. B: Mean OS in the Caveolin-1 positive and the Caveolin-1 negative group was 87 months (95%CI 79–96) and 84 months (95%CI 70–98; p = 0.72), respectively.

There were no significant differences between Caveolin-1 positive and Caveolin-1-negative cases with respect to survival. Mean DFS was 78 months (95%CI 67–95) in Caveolin-1 positive as compared to 82 months (95%CI 72–93) in Caveolin-1 negative patients (p = 0.66) (Figure 2A). Mean OS was 87 months (95%CI 79–96) in the Caveolin-1 positive and 84 months (95%CI 70–98) in the Caveolin-1 negative group (p = 0.72) (Figure 2B). Subgroup analysis according to clinical and pathological parameters as well as to different treatment did not reveal any prognostic significance of Caveolin-1 expression (data not shown).

## Discussion

Since its first description as a major v-Src-substrate in Rous sarcoma virus-transformed chicken embryo fibroblasts, Caveolin-1 has been considered as a presumable mediator of transformation by oncogenic tyrosine kinases [22]. The contribution of Caveolin-1 to carcinogenesis and tumour progression has been intensively evaluated.

In order to examine the potential clinical relevance of Caveolin-1 in premalignant and malignant breast disease, we studied Caveolin-1 protein expression in tissue probes of healthy breast tissue, benign breast disease, DCIS, and invasive breast cancer, using immunohistochemistry. We found Caveolin-1 expression in epithelial tumour cells in 32 of 109 cases (29.4%) of invasive breast carcinomas. In contrast, when evaluating 108 cases of DCIS specimens, 236 cases of benign breast disease and five cases of healthy breast tissue, no Caveolin-1 expression could be found in the epithelial component. Caveolin-1 expression was consistently detected in ductal and lobular myoepithelial cells, in vascular smooth muscle cells, and in endothelial cells in non-malignant breast tissue samples, which is in concordance with previous reports [14,21].

Several comprehensive immunohistochemical studies have reported on Caveolin-1 expression in human breast cancer; Yang *et al*.[23], examined Caveolin-1 protein expression in 15 cases of invasive breast cancer, 15 cases of intraductal breast cancer, and 9 cases of lymph node metastasis. They reported significantly higher expression of Caveolin-1 in both intraductal carcinomas (p > 0.001) and infiltrating ductal carcinomas (93.3%, p < 0.001) as well as in lymph node metastases (p < 0.001) relative to normal breast epithelium. However, even in normal breast epithelial cells, minimal staining was observed. In contrast, Hurlstone *et al*.[14], supported our observations in that they could not detect Caveolin-1 expression within the epithelial cell component of human mammary normal ducts or terminal ductal lobular units of 10 breast reduction specimens. Instead, high Caveolin-1 expression levels were again observed in mammary myoepithelial cells. In the most recent immunohistochemical study, Savage *et al*.[21], studied the frequency and cellular distribution of Caveolin-1 expression in normal breast, benign breast lesions, breast cancer precursors, and breast carcinomas. Using a monoclonal antibody, the authors corroborated our results in that no expression of Caveolin-1 could be observed in the epithelial cell component of normal breast tissue or in luminal epithelial cells of benign breast lesions such as radial scars. However, luminal epithelial cells demonstrated Caveolin-1 expression in 13.4% of DCIS and 9.4% of invasive breast cancer specimens. The authors observed an inverse correlation between Caveolin-1 expression and expression of ER, PR, HER2, and cyclin D1, as well as an association with the expression of EGFR, cytokeratins 5/6, 14, and 17, high MIB-1 expression, and p53 expression. Furthermore, they described a significant association between Caveolin-1 expression and both shorter disease-free and overall survival as well as with the so-called 'basal-like' immunophenotype, which also has been repeatedly associated with adverse clinical outcome [21]. Interestingly, an association between basal-like phenotype and Caveolin-1 expression has been described in another report. Pinilla *et al*.[24], examined Caveolin-1 expression in 509 cases of sporadic and 47 cases of hereditary breast cancers using a monoclonal Caveolin-1 antibody. Caveolin-1 expression was observed among 4.6% of sporadic cases, but among as many as 10.6% of hereditary cases. Caveolin-1 positivity was again significantly associated with lack of ER, PR, and HER2 expression and presence of cytokeratin 5/6 and EGFR expression. Lack of expression of ER and HER2 expression and presence of cytokeratin 5/6 and/or EGFR expression were taken as surrogate markers indicating a basal-like phenotype. Accordingly, 52% of Caveolin-1 positive cases were classified as basal-like subtype. These results are in striking contrast to observation by Sagara *et al*., The group examined 162 breast cancer specimens using the same monoclonal anti-Caveolin-1 antibody and realtime-PCR. They described a significant positive correlation between Caveolin-1-mRNA expression in breast cancer and positive oestrogen receptor-status as well as reduced tumour size [20]. In our study, neither oestrogen receptor status, nor tumour stage, nor other clinical or pathological parameters, besides multifocality (p = 0.008), correlated with Caveolin-1 expression. Furthermore, no significant correlation with either disease free survival or overall survival could be demonstrated.

Choice of primary antibody and scoring system has been shown to have a substantial impact on the results of immunoreactivity. For example, Kersting *et al*., determined epidermal growth factor receptor (EGFR) immunoreactivity in 302 cases of soft tissue sarcomas using five different commercially available antibodies, and EGFR amplification status in 283 cases using fluorescence in situ hybridisation (FISH). Depending on the antibody and scoring method used, EGFR expression frequency varied between 0.3% and 52.9%. EGFR gene amplification was determined in 3.5% of tumours showed and correlated with EGFR expression for only three antibodies [25]. Of note, Yang *et al*., used a polyclonal antiserum to determine Caveolin-1 expression status in human prostate and breast malignancies. They reported positive staining in as many as 80% of cases of intraductal carcinomas and also minimal Caveolin-1 expression in normal breast epithelium. In contrast, when using a monoclonal antibody, Hurlstone *et al*., corroborated our results, in that no Caveolin-1 expression was observed among normal breast epithelial cells [14]. Different scoring methods might explain some of the discrepancies between our results and those of Savage *et al*.[21]. The group applied a semiquantitative consensus score of both distribution and intensity of Caveolin-1 immunostaining. Based on a cutoff score of ≥ 4 they reported Caveolin-1 expression in 9.4% of primary breast cancers. In our study we applied a 4-tired semiquantitative score to describe intensity of Caveolin-1 expression. In face of the lack of Caveolin-1 expression in normal breast epithelial cells, we considered any Caveolin-1 staining as positive and combined weak, moderate and strong Caveolin-1 expression (scores "1" to "3") to represent Caveolin-1 positivity. Thus, we observed Caveolin-1 expression in 32 of 109 cases of invasive breast carcinomas (29.4%). However, if we had only regarded moderate and strong expression, we would have observed Caveolin-1 expression in only 7.3% of cases, which is in the range of the results by Savage *et al.*[21].

Interestingly, Savage *et al*., reported Caveolin-1 immunostaining in 2 of 15 cases (13.4%) of DCIS [21]. This is in striking contrast to our results. We examined 108 cases of DCIS and could not find Caveolin-1 positivity among these cases. In concordance with the methodology of Savage *et al*., entrapped blood vessels were used as internal positive controls in order to ensure robustness of the data. The TMAs in this study included endothelial cells in both malignant tumour specimens as well as adjacent normal breast tissue. Endothelial cells were consistently found to be Caveolin-1 positive.

The role of Caveolin-1 in mammary carcinogenesis is still far from being completely understood. Scientific evidence of a tumour suppressive role of Caveolin-1 in breast cancer supported by some researchers [26] is contrasted by recent results which strengthen the role of Caveolin-1 overexpression to promote certain steps of tumourigenesis: Caveolin-1 has been shown to inhibit anoikis in MCF7 breast cancer cells [27]. Furthermore, Caveolin-1 has been demonstrated to mediate medroxyprogesterone acetate-(MPA)-induced breast cancer cell growth [28]. Inflammatory breast cancer represents a highly aggressive form of invasive breast cancer. Among these cancers, Caveolin-1 expression is upregulated compared to expression levels in non-inflammatory carcinomas [29]. In face of this controversy one has to assume that the role of Caveolin-1 as both tumour suppressor and promoter might be context-depending. While being downregulated in early stage malignancies and thereby mediating growth promoting effects, upregulation of Caveolin-1 in late stage disease might promote resistance against chemotherapeutic agents in colon cancer as well as metastatic properties in prostate cancer [30]. It seems reasonable that both the conflicting data on Caveolin-1 expression frequencies and the lack of a clear prognostic impact in breast cancer mirror the variety of functions, which Caveolin-1 is believed to obtain in breast cancer pathogenesis. Caveolin-1 has been shown to determine the function of caveolae as a platform to preassemble distinct components of cellular pathways, and therefore both to render signal transduction more efficient and to enable appropriate interaction between distinct pathways [31]. This allows placing the protein components in close proximity to each other. Thus, its distinct role in cellular processes may depend on the combination of proteins expressed in the cells rather than on Caveolin-1 expression itself.

Importantly, recent results have revealed a potential therapeutic relevance of Caveolin-1 since the Caveolin-1-promoter has been hypothesized to be used as a specific target in gene therapy of prostate carcinoma in the nearer future [32]. Bortezomib, an antibody against the 26-S-proteasome, has been shown to target Caveolin-1 among a variety of other proteins in studies in various cancer entities [33].

## Conclusion

In our immunohistochemical study, we found significant Caveolin-1 expression in one third of invasive breast carcinomas, whereas neither normal breast tissue, nor benign breast disease, nor DCIS showed relevant Caveolin-1 expression. Furthermore, we demonstrated that Caveolin-1 expression alone does not show any clear-cut prognostic or predictive properties. We hypothesize that instead of being an independent prognostic factor alone, Caveolin-1 might exhibit a more complex function that needs to be evaluated in context with the co-expressed proteins as well as in view of the respective disease stage. This might finally explain the conflicting results described in the scientific literature. Further studies are warranted to understand the role of Caveolin-1 expression in the disease course of breast cancer as well as its potential as a therapeutic target.

## Competing interests

The author(s) declare that they have no competing interests.

## Authors' contributions

CL designed and coordinated the study, and drafted the manuscript.

CK performed the TMA read-out and scoring and assisted in drafting the manuscript.

HB participated in the coordination of the study.

LK participated in design of the study.

PW designed the TMA, performed the statistical analysis, and critically reviewed the manuscript.

All authors read and approved the final manuscript.

## References

[B1] Parkin DM, Bray F, Ferlay J, Pisani P (2005). Global cancer statistics, 2002. CA Cancer J Clin.

[B2] Fielding CJ, Fielding PE (2000). Cholesterol and caveolae: structural and functional relationships. Biochim Biophys Acta.

[B3] Li S, Okamoto T, Chun M, Sargiacomo M, Casanova JE, Hansen SH, Nishimoto I, Lisanti MP (1995). Evidence for a regulated interaction between heterotrimeric G proteins and caveolin. J Biol Chem.

[B4] Song KS, Li S, Okamoto T, Quilliam LA, Sargiacomo M, Lisanti MP (1996). Co-purification and direct interaction of Ras with caveolin, an integral membrane protein of caveolae microdomains. J Biol Chem.

[B5] Garcia-Cardena G, Martasek P, Masters BS, Skidd PM, Couet J, Li S, Lisanti MP, Sessa WC (1997). Dissecting the interaction between nitric oxide synthase (NOS) and caveolin. Functional significance of the nos caveolin binding domain in vivo. J Biol Chem.

[B6] Lisanti MP, Scherer PE, Tang Z, Sargiacomo M (1994). Caveolae, caveolin and caveolin-rich membrane domains: a signalling hypothesis. Trends Cell Biol.

[B7] Koleske AJ, Baltimore D, Lisanti MP (1995). Reduction of caveolin and caveolae in oncogenically transformed cells. Proc Natl Acad Sci USA.

[B8] Suzuoki M, Miyamoto M, Kato K, Hiraoka K, Oshikiri T, Nakakubo Y, Fukunaga A, Shichinohe T, Shinohara T, Itoh T, Kondo S, Katoh H (2002). Impact of caveolin-1 expression on prognosis of pancreatic ductal adenocarcinoma. Br J Cancer.

[B9] Yoo SH, Park YS, Kim HR, Sung SW, Kim JH, Shim YS, Lee SD, Choi YL, Kim MK, Chung DH (2003). Expression of caveolin-1 is associated with poor prognosis of patients with squamous cell carcinoma of the lung. Lung Cancer.

[B10] Campbell L, Gumbleton M, Griffiths DF (2003). Caveolin-1 overexpression predicts poor disease-free survival of patients with clinically confined renal cell carcinoma. Br J Cancer.

[B11] Yang G, Truong LD, Wheeler TM, Thompson TC (1999). Caveolin-1 expression in clinically confined human prostate cancer: a novel prognostic marker. Cancer Res.

[B12] Engelman JA, Lee RJ, Karnezis A, Bearss DJ, Webster M, Siegel P, Muller WJ, Windle JJ, Pestell RG, Lisanti MP (1998). Reciprocal regulation of neu tyrosine kinase activity and caveolin-1 protein expression in vitro and in vivo. Implications for human breast cancer. J Biol Chem.

[B13] Zenklusen JC, Bieche I, Lidereau R, Conti CJ (1994). (C-A)n microsatellite repeat D7S522 is the most commonly deleted region in human primary breast cancer. Proc Natl Acad Sci USA.

[B14] Hurlstone AF, Reid G, Reeves JR, Fraser J, Strathdee G, Rahilly M, Parkinson EK, Black DM (1999). Analysis of the CAVEOLIN-1 gene at human chromosome 7q31.1 in primary tumours and tumour-derived cell lines. Oncogene.

[B15] Wulfing P, Diallo R, Kersting C, Wulfing C, Poremba C, Rody A, Greb RR, Bocker W, Kiesel L (2003). Expression of endothelin-1, endothelin-A, and endothelin-B receptor in human breast cancer and correlation with long-term follow-up. Clin Cancer Res.

[B16] Wulfing P, Diallo R, Muller C, Wulfing C, Poremba C, Heinecke A, Rody A, Greb RR, Bocker W, Kiesel L (2003). Analysis of cyclooxygenase-2 expression in human breast cancer: high throughput tissue microarray analysis. J Cancer Res Clin Oncol.

[B17] Holland R, Peterse JL, Millis RR, Eusebi V, Faverly D, van de Vijver MJ, Zafrani B (1994). Ductal carcinoma in situ: a proposal for a new classification. Semin Diagn Pathol.

[B18] Wulfing P, Kersting C, Buerger H, Mattsson B, Mesters R, Gustmann C, Hinrichs B, Tio J, Bocker W, Kiesel L (2005). Expression patterns of angiogenic and lymphangiogenic factors in ductal breast carcinoma in situ. Br J Cancer.

[B19] Kononen J, Bubendorf L, Kallioniemi A, Barlund M, Schraml P, Leighton S, Torhorst J, Mihatsch MJ, Sauter G, Kallioniemi OP (1998). Tissue microarrays for high-throughput molecular profiling of tumor specimens. Nat Med.

[B20] Sagara Y, Mimori K, Yoshinaga K, Tanaka F, Nishida K, Ohno S, Inoue H, Mori M (2004). Clinical significance of Caveolin-1, Caveolin-2 and HER2/neu mRNA expression in human breast cancer. Br J Cancer.

[B21] Savage K, Lambros MB, Robertson D, Jones RL, Jones C, Mackay A, James M, Hornick JL, Pereira EM, Milanezi F, Fletcher CD, Schmitt FC, Ashworth A, Reis-Filho JS (2007). Caveolin 1 is overexpressed and amplified in a subset of basal-like and metaplastic breast carcinomas: a morphologic, ultrastructural, immunohistochemical, and in situ hybridization analysis. Clin Cancer Res.

[B22] Glenney JR (1989). Tyrosine phosphorylation of a 22-kDa protein is correlated with transformation by Rous sarcoma virus. J Biol Chem.

[B23] Yang G, Truong LD, Timme TL, Ren C, Wheeler TM, Park SH, Nasu Y, Bangma CH, Kattan MW, Scardino PT, Thompson TC (1998). Elevated expression of caveolin is associated with prostate and breast cancer. Clin Cancer Res.

[B24] Pinilla SM, Honrado E, Hardisson D, Benitez J, Palacios J (2006). Caveolin-1 expression is associated with a basal-like phenotype in sporadic and hereditary breast cancer. Breast Cancer Res Treat.

[B25] Kersting C, Packeisen J, Leidinger B, Brandt B, von Wasielewski R, Winkelmann W, van Diest PJ, Gosheger G, Buerger H (2006). Pitfalls in immunohistochemical assessment of EGFR expression in soft tissue sarcomas. J Clin Pathol.

[B26] Williams TM, Medina F, Badano I, Hazan RB, Hutchinson J, Muller WJ (2004). Caveolin-1 gene disruption promotes mammary tumorigenesis and dramatically enhances lung metastasis in vivo. Role of Cav-1 in cell invasiveness and matrix metalloproteinase (MMP-2/9) secretion. J Biol Chem.

[B27] Fiucci G, Ravid D, Reich R, Liscovitch M (2003). Caveolin-1 inhibits anchorage-independent growth, anoikis and invasiveness in MCF-7 human breast cancer cells. Oncogene.

[B28] Salatino M, Beguelin W, Peters MG, Carnevale R, Proietti CJ, Galigniana MD (2006). Progestin-induced caveolin-1 expression mediates breast cancer cell proliferation. Oncogene.

[B29] Van den Eynden GG, Van Laere SJ, Van der Auwera I, Merajver SD, Van Marck EA, van Dam P (2006). Overexpression of caveolin-1 and -2 in cell lines and in human samples of inflammatory breast cancer. Breast Cancer Res Treat.

[B30] Shatz M, Liscovitch M (2004). Caveolin-1 and cancer multidrug resistance: coordinate regulation of pro-survival proteins?. Leuk Res.

[B31] Marx J (2001). Caveolae: a once-elusive structure gets some respect. Science.

[B32] Pramudji C, Shimura S, Ebara S, Yang G, Wang J, Ren C, Yuan Y, Tahir SA, Timme TL, Thompson TC (2001). In situ prostate cancer gene therapy using a novel adenoviral vector regulated by the caveolin-1 promoter. Clin Cancer Res.

[B33] Boccadoro M, Morgan G, Cavenagh J (2005). Preclinical evaluation of the proteasome inhibitor bortezomib in cancer therapy. Cancer Cell Int.

